# Prognostic value of the sliding length of cephalocervical screws to predict the risk of non-union after osteosynthesis: a retrospective analysis of 86 patients with intracapsular femoral neck fractures

**DOI:** 10.1186/s13018-017-0533-z

**Published:** 2017-02-22

**Authors:** Yohei Hirakawa, Hidehiro Nakamura, Kazuhito Minamitani, Ryuki Hashida, Masafumi Gotoh, Naoto Shiba

**Affiliations:** 1 Department of Orthpaedics, Munakata Suikokai General Hospital, 5-7-1 Himakino, Fukutu, Fukuoka 811-3298 Japan; 2 Department of Orthopaedics, Kurume Unversity, 67 Asahi-machi, Kurume, Fukuoka 830-0011 Japan; 3 Department of Orthopaedics, Kurume University Medical Center, 155-1 Kokubu-machi, Kurume, Fukuoka 839-0863 Japan

**Keywords:** Cephalocervical screw, Femoral neck fracture, Internal fixation

## Abstract

**Background:**

Here, we assessed the prognostic value of the early sliding length (ESL) for predicting the risk of non-union after internal fixation of femoral neck fractures (FNFs) by Dual SC Screws (DSCS).

**Methods:**

A retrospective analysis of 86 patients with intra-capsular FNFs was performed. They underwent osteosynthesis by DSCS at our institution between 2008 and 2013 with a minimum follow-up duration of 6 months. Preoperative displacement, fracture reduction quality, ESL of screws at 2 weeks postoperatively, and correlation of non-union with the ESL of screws were evaluated.

**Results:**

Bone union without complications was achieved in 74 patients (86.0%), whereas 12 patients (14.0%) showed non-union. The ESL was significantly longer in the non-union group (proximal 3.94 ± 2.79 mm, distal 4.03 ± 3.16 mm) than in the union group (proximal 0.98 ± 1.85 mm, distal 1.01 ± 1.84 mm, *P* = 0.0001* for proximal, *P* < 0.0001* for distal). The ESL was significantly associated with non-union, both in the proximal [*P* = 0.0002, unit odds ratio (OR) 1.58, 95% confidence interval (CI) 1.23–2.16] and distal screws (*P* = 0.0002, unit OR 1.53, 95% CI 1.21–2.02). The areas under the ROC curves for the ESL of proximal and distal screws were 0.845 and 0.867, respectively; the cut-off values to predict non-union were 1.0 mm (sensitivity 91.7% and specificity 74.3%) and 1.4 mm (sensitivity 83.3% and specificity 81.1%), respectively.

**Conclusions:**

In this study, the ESL was a good predictor of postoperative non-union in patients with FNFs fixed by DSCS.

## Background

Intracapsular femoral neck fracture (FNF) is a very common orthopedic injury among the elderly [1, 2]. With increasing average life expectancy, the incidence of these fractures is steadily increasing worldwide [1–3]. Internal fixation (IF) is the preferred treatment for undisplaced FNF [1, 4]. Operative alternatives for FNFs in young adults are still being debated; the available options include prosthetic replacement (arthroplasty) and IF [1, 5, 6]. In either case, prompt operative treatment and immediate postoperative weight bearing are key tenets of treatment of FNFs for optimal functional restoration.

Recent studies indicate improved rates of the union of FNFs with the use of cephalocervical screws with an inbuilt sliding mechanism [7–9]. The sliding mechanism on IF allows dynamic interfragmentary compression postoperatively by the load of weight bearing [7]. Typically, this dynamic interfragmentary compression system promotes osteosysthesis. However, too early and/or excessive sliding of the neck may result in non-union. The criteria for excessive sliding are not well elucidated.

The purpose of the present study was to assess the correlation between the non-union rate and postoperative sliding length in patients with FNFs treated with cephalocervical screws. The cut-off values of the sliding length of cephalocervical screws to predict the risk of non-union were calculated. The study hypothesis is that the postoperative sliding length immediately after weight bearing has a significant correlation with non-union.

## Methods

This retrospective study was approved by an authorized institutional review board (Kurume University, Japan, no.14173).

### Subjects

Between December 2008 and December 2013, 96 patients with femoral neck fracture were treated surgically with Dual SC Screws (DSCS) [10] at our hospital (Munakata Suikokai General Hospital, Japan). The inclusion criteria were as follows: (1) weight bearing initiated within 2 weeks after the surgery; (2) availability of postoperative radiographs at three time-points (immediate postoperative period; 2 weeks after surgery; final follow-up); and (3) minimum duration of follow-up of 6 months.

The exclusion criteria were as follows: (1) patients with pathological fractures and open fractures and (2) those with postoperative infections or severe complications that did not allow initiation of weight bearing within 2 weeks after the surgery.

In total, 53 fractures were classified as undisplaced (Garden stages I and II) and 33 fractures as displaced (Garden stages III and IV). All fractures were treated using a traction table and fluoroscopy without any open reduction. At first, we longitudinally pulled the lower limbs and gradually performed abduction, and a hip joint was internally rotated gently for good reduction of the displaced fractures.

### Operative technique and rehabilitation protocol

All operations were performed using a radiolucent traction table under fluoroscopic guidance using standard procedures outlined below [7]. A 4-cm-long lateral incision was made directly through the skin to the bone; a guide wire was inserted from the lateral cortex (at the level for lesseor trochanter) to the femoral head [inferior 1/2 area in anteroposterior (AP) view] at an approximate angle of 135°. As the first guide wire was inserted, fluoroscopy was used to confirm satisfactory alignment, close to the calcar on the AP view and centrally on the lateral view. A second guide wire was inserted to the superior 1/2 area of the femoral head in a parallel direction to the first guide wire, temporarily before reaming to avoid proximal fragment rotation. The second guide wire should rest on the dorsal cortex on lateral view, and the first and second wires spread as far as possible on the AP view. After reaming, the lag screws were introduced. The screws were parallel in both planes, and the tip of each screw was driven to within 5 mm of the subchondral bone plate. All patients were permitted weight bearing with crutches/walker assistance within at least 2 weeks after surgery to prevent long-term bed rest.

### Clinical assessment

All patients were retrospectively evaluated using medical records. Data pertaining to the following variables were extracted: age, sex, waiting time for surgery (days), operation time (min), blood loss (g), duration of follow-up (months), and pre-/postoperative walking ability.

### Assessment of radiographs

The AP view of the hip was obtained with the patients in the supine position and the lower limbs in a medial rotation of 15°. The radiographic magnification was determined by dividing the known diameter of the lag screw with the diameter measured on the radiographs [7]. The Garden alignment index was calculated from the immediate postoperative radiograph [11]. The length of the proximal/distal screws was measured at each time point. The early sliding length (ESL) after weight bearing was measured by comparing the AP radiographs obtained at the first and second time-points; the final sliding length (FSL) was measured by comparing the AP radiographs obtained at the first and third time-points. Non-union was defined at 6 months postoperatively as re-displacement/an absence of radiographically visible trabeculae across the fracture line [9]. Radiographic evaluation was performed by a senior orthopedician who was blinded to the study.

### Statistical analysis

All statistical processing was performed with JMP11 (SAS, Cary, NC). The Mann–Whitney *U* test and *χ* ^2^ test were used to compare continuous and nominal variables between union and non-union groups, respectively. The effect of ESL/FSL of proximal/distal screw on non-union was analyzed by logistic regression. Receiver operating characteristic (ROC) curves were generated for the ESL/FSL as the predictor of non-union. The cut-off point was determined by the area under the ROC curve. *P* < 0.05 indicated statistical significance for all analyses.

## Results

Overall, 86 patients [18 men and 68 women, mean age 81.9 ± 10.3 (range 54–99) years] fulfilled the inclusion criteria and were enrolled in the study. Ten patients were excluded [rehabilitation protocol was not followed because of complications (*n* = 2), follow-up duration < 6 months (*n* = 8)]. Mean waiting time before surgery was 4.9 ± 3.6 (range 1–17) days, the mean intraoperative blood loss was 20.6 ± 13.6 (range 0–100) g, and the mean duration of follow-up was 12.5 ± 10.5 (range 6–48) months.

**Fig. 1:**
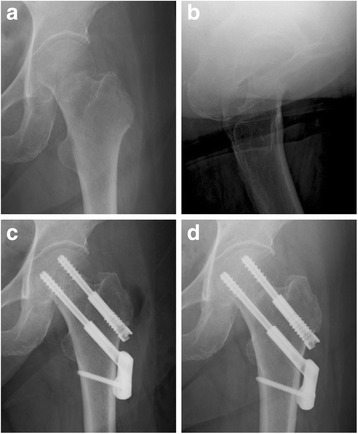
Plain radiographs demonstrating a femoral neck fracture fixed with Dual SC Screws. The screws were positioned adequately, and the bone union was obtained with short sliding length. (**a**, **b** preoperative, **c** postoperative, **d** 6 months postoperatively)

**Fig. 2:**
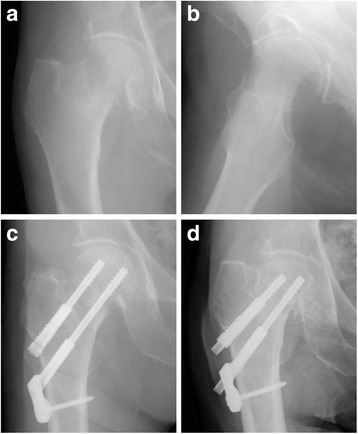
Radiographs showing a case of non-union of femoral neck fracture after internal fixation. Excessive sliding of Dual SC Screws was observed. (**a**, **b** preoperative, **c** postoperative, **d** ;4 months postoperatively)

Based on the evaluation of postoperative X-ray radiographs obtained at a minimum of 6 months from the date of surgery, 74 patients (86.0%) were in the union group (Fig. 1) and 12 (14%) were in the non-union group (Fig. 2). In the non-union group, 11 patients underwent secondary hemiarthroplasty, while no additional operation was performed in one patient because of complications. As a limitation, avascular necrosis was not observed during the short observation period in this study. Further observation is required to determine the occurrence of this complication.

The baseline characteristics of patients by study group are presented in Table 1. No significant between-group differences were observed with respect to demographic variables, including age, sex, waiting time, operative blood loss, duration of follow-up, preoperative ADL, and postoperative Garden alignment index (both in AP and lateral view). Significant between-group difference was observed with respect to the Garden classification and the postoperative ADL.

**Table 1:** Baseline characteristics of patients according to the study group

	Union group (*n* = 74)	Non-union group (*n* = 12)	*P* value
Age (years)	81.4 ± 10.4 (54–99)	85.3 ± 9.7 (62–96)	0.1606
Sex (male/female)	17 (23.0%)/57 (77.0%)	1 (8.3%)/11 (91.7%)	0.2475
Waiting time (days)	5.0 ± 3.6 (1–17)	4.4 ± 3.3 (1–12)	0.5339
Intraoperative blood loss (g)	21.0 ± 14.2 (0–100)	17.9 ± 9.9 (5–40)	0.5756
Duration of follow-up (m)	13.0 ± 11.0 (6–48)	9.0 ± 6.2 (6–26)	0.1826
Garden classification			
	Stage I: 8 (10.8%)	Stage I: 0 (0%)	0.0008^a^
	Stage II: 44 (59.5%)	Stage II: 1 (8.3%)	
	Stage III: 18 (24.3%)	Stage III: 9 (75.0%)	
	Stage IV: 4 (5.4%)	Stage IV: 2 (16.7%)	
Garden alignment index			
AP view	168.0 ± 6.2	164.5 ± 8.6	0.168
Lateral view	173.4 ± 5.3	169.7 ± 10.2	0.159
Preoperative ADL			
Gait alone	31(41.9%)	4(33.3%)	0.4917
With aid	18(24.3%)	2(16.7%)	
With helper/walker	21(28.4%)	4(33.3%)	
Wheel chair	4(5.4%)	2(16.7%)	
Postoperative ADL			
Gait alone	5(6.8%)	0(0%)	0.0493^a^
With aid	33(44.6%)	1(8.3%)	
With helper/walker	18(24.3%)	5(41.7%)	
Wheel chair	18(24.3%)	6(50.0%)	

Note. Data presented as mean ± standard deviation unless indicated otherwise

*AP* anteroposterior, *ADL* activities of daily living

^a^ *P* < 0.05 among the three groups with *χ* ^2^ test

The rate of unstable fracture (Garden stages III and IV) in the union group (29.7%) was significantly lower than that in the non-union group (91.7%) (*P* = 0.0008*). Postoperative ADL was significantly superior in the union group than that in the non-union group (*P* = 0.0493*).

The FSL of DSCS in the non-union group was significantly longer (proximal 9.66 ± 6.52 mm, distal 10.94 ± 4.57 mm) than that in the union group (proximal 2.92 ± 3.94 mm, distal 3.34 ± 4.13 mm) (*P* = 0.0045 for proximal; *P* < 0.0001* for distal). Furthermore, the ESL at 2 weeks after surgery was significantly longer in the non-union group (proximal 3.94 ± 2.79 mm, distal 4.03 ± 3.16 mm) than that in the union group (proximal 0.98 ± 1.85 mm, distal 1.01 ± 1.84 mm) (*P* = 0.0001* for proximal, *P* < 0.0001* for distal) (Tables 2, 3, and 4).

**Table 2:** Final sliding length of proximal and distal screws

	Union (*n* = 74)	Non-union (*n* = 12)	*P* value
Proximal	2.93 ± 3.94 (0–19.9)	9.66 ± 6.53 (0.1–17.9)	0.0045^b^
Distal	3.34 ± 4.13 (0–21.6)	10.94 ± 4.57 (2.6–17.3)	<0.0001^b^

Note. Data presented as mean ± standard deviation unless otherwise indicated

^b^Statistically significant (*P* < 0.05) among the three groups using the Mann–Whitney *U* test

**Table 3:** Early sliding length of proximal and distal screws at 2 weeks after surgery

	Union (*n* = 74)	Non-union (*n* = 12)	*P* value
Proximal	0.98 ± 1.85 (0–11.8)	3.94 ± 2.79 (0–10.2)	0.0001^b^
Distal	1.01 ± 1.84 (0–9.8)	4.03 ± 3.16 (0–11.5)	<0.0001^b^

Note. Data presented as mean ± standard deviation unless otherwise indicated

^b^Statistically significant (*P* < 0.05) among the three groups using the Mann–Whitney U test

**Table 4:** Cut-off value of the final sliding length for predicting the risk of non-union

	AUC	Cut-off value	*P* value	*Sensitivity*	*Specificity*
Proximal	0.75676	10.9 mm	<0.0001*	0.5991	0.9324
Distal	0.89752	7.6 mm	<0.0001*	0.6982	0.8649

Note. *P* value was calculated using logistic regression. Receiver operating characteristic (ROC) curves were generated to detect the cut-off value of sliding length for predicting the risk of non-union. *AUC* area under the curve

*P < 0.05 was considered indicative of *statistical significance.

**Fig. 3:**
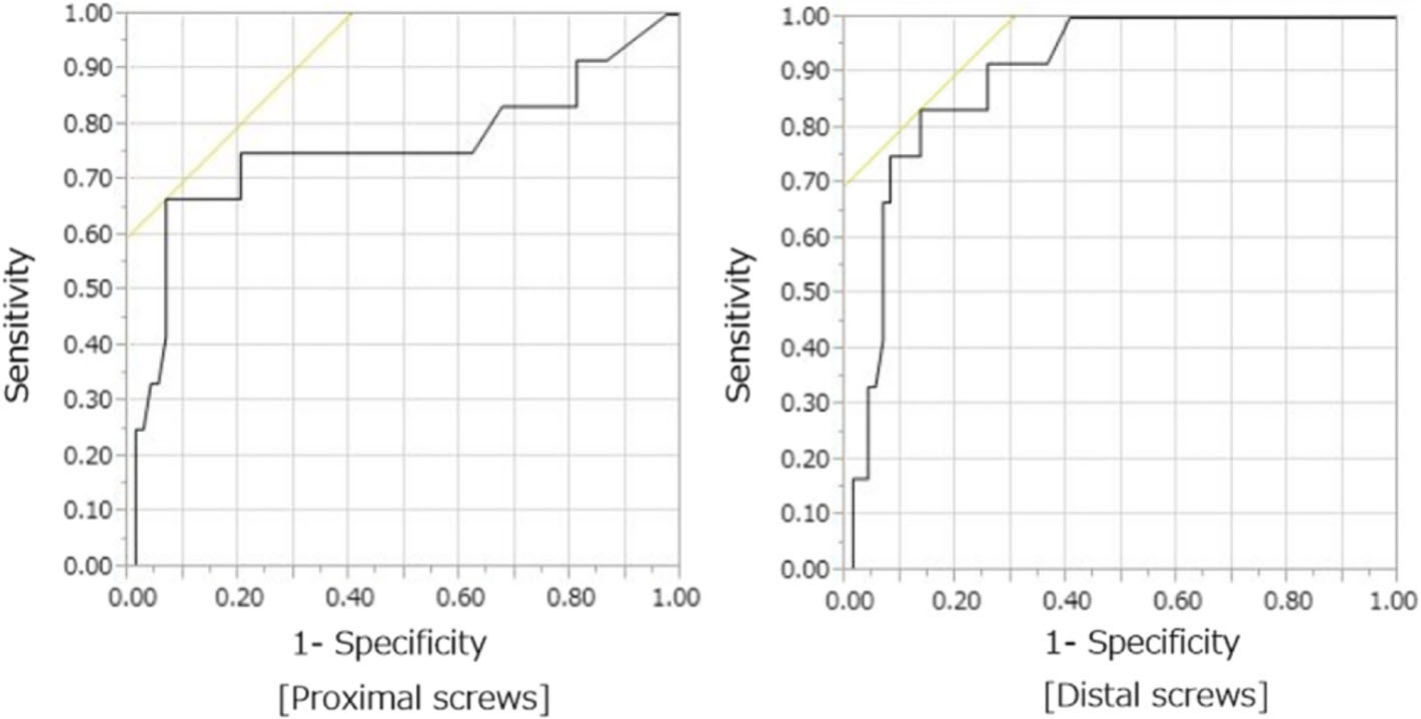
Receiver operating characteristic (ROC) curves for the final sliding length of proximal and distal screws. Areas under the ROC curve were 0.757 and 0.898, respectively

On logistic regression analysis, the FSL of proximal screw [*P* < 0.0001, unit odds ratio (OR) 1.25, 95% confidence interval (CI) 1.12–1.42] and that of distal screw (*P* < 0.0001, unit OR 1.31, 95% CI 1.16–1.52) were significantly associated with non-union. The areas under the ROC curves for the FSL of proximal and distal screws were 0.757 and 0.898, respectively, and the cut-off values to predict non-union were 10.9 (sensitivity 66.7% and specificity 93.2%) and 7.6 mm (sensitivity 69.8% and specificity 86.5%), respectively (Fig. 3 and Table 4).

**Fig. 4:**
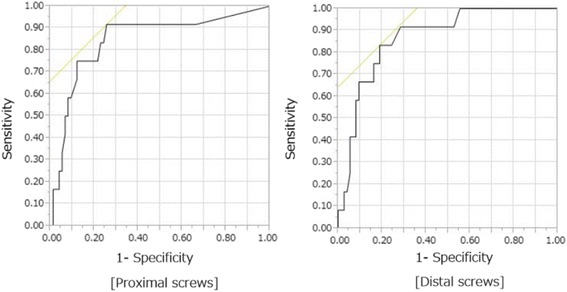
Receiver operating characteristic (ROC) curves for the early sliding length of proximal and distal screws. Areas under the ROC curve were 0.845 and 0.867, respectively

On logistic regression analysis, the ESL of proximal screws was significantly associated with non-union (*P* = 0.0002, unit OR 1.58, 95% CI 1.23–2.16), while the ESL of distal screws was significantly associated with non-union (*P* = 0.0002, unit OR 1.53, 95% CI 1.21–2.02). The areas under the ROC curves for the ESL of proximal and distal screws were 0.845 and 0.867, respectively, and the cut-off values to predict non-union were 1.0 (sensitivity 91.7% and specificity 74.3%) and 1.4 mm (sensitivity 83.3% and specificity 81.1%), respectively (Fig. 4 and Table 5).

**Table 5:** Cut-off values of the early sliding length for predicting the risk of non-union

	AUC	Cut-off value	*P* value	*Sensitivity*	*Specificity*
Proximal	0.84459	1.0 mm	0.0002*	0.9167	0.7432
Distal	0.86712	1.4 mm	0.0002*	0.8333	0.8109

Note. *P* value was calculated using logistic regression. Receiver operating characteristic (ROC) curves were generated to detect the cut-off value of sliding length for predicting the risk of non-union. *AUC* area under the curve

*P < 0.05 was considered indicative of *statistical significance

## Discussion

This study showed that the postoperative sliding length immediately after weight bearing (ESL) is a good predictor of postoperative non-union in patients with FNFs fixed by cephalocervical screws, and the cut-off ESL values were 1.0 mm for the proximal screw and 1.4 mm for the distal screw.

Internal fixation is the conventional treatment of choice for undisplaced FNFs in the elderly and displaced fractures in young adults. It enables earlier mobility with complete weight bearing and avoidance of prolonged recumbence. Several methods are available for the internal fixation of FNFs: compression screws, dynamic condylar screws, locked plates, and sliding hip screws [12, 13]. The DSCS, (KISCO DIR Co., Ltd., Kobe) used in this study is also helpful for the treatment of intracapsular hip fractures. It consists of two cephalocervical screws in a sliding mechanism that allows linear postoperative compression that may promote fracture union, and enables early postoperative mobility with complete weight bearing. Nishiyama et al. reported good clinical outcomes in patients with FNFs fixed by DSCS [7].

Previous studies have reported some predictors of non-union after the fixation of intracapsular FNFs; however, these include poor reduction and fracture displacement [10, 14–16]. The type of fixation may also predict early failure after operation. Gardner et al. reported that the use of sliding hip screw constructs was associated with a significantly lower short-term failure rate than that with cannulated screw constructs in patients with displaced FNFs [17]. Dolatowski et al. reported that a preoperative posterior tilt of at least 20° increased the risk of fixation failure in stable FNFs [18]. However, to the best of our knowledge, the correlation between the sliding length of screws and the rate of non-union has not been reported.

The relatively small sample size limits the statistical power of our analyses. In addition, follow-up and measurement bias cannot be excluded because of the retrospective study design. Larger prospective studies are required for a more definitive evaluation of outcomes.

## Conclusions

In this study, we observed a significant correlation between the ESL of screws and the incidence of non-union following the treatment of FNF with DSCS.

## Abbreviations

AP: Anteroposterior
CI: Confidence interval
DSCS: Dual SC Screws
ESL: Early sliding length
FNFs: Femoral neck fractures
FSL: Final sliding length
IF: Internal fixation
OR: Odds ratio
ROC: Receiver operating characteristic

## References

[1] Gjertsen JE, Fevang JM, Martre K, Vinje T, Engesæter LB (2011). Clinical outcome after femoral neck fractures. Acta Prthop.

[2] Miyamoto RG, Kaplan KM, Levine BR, Egol KA, Zuckerman JD (2008). Surgical management of hip fractures: an evidence-based review of the literature. I: femoral neck fractures. J Am Acad Orthop Surg.

[3] Simonen O (1991). Incidence of femoral neck fractures: senile osteoporosis in Finland in the years 1970–1985. Calcif Tissue Int.

[4] Hongisto MT, Pihlajamäki H, Niemi S, Nuotio M, Kannus P, Mattila VM (2014). Surgical procedures in femoral neck fractures in Finland: a nationwide study between 1998 and 2011. Int Orthop.

[5] Blomfeldt R, Tornkvist H, Ponzer S, Söderqvist A, Tidermark J (2005). Internal fixation versus hemiarthroplasty for displaced fractures of the femoral neck in elderly patients with severe cognitive impairment. J Bone Joint Surg (Br).

[6] Frihagen F, Nordsletten L, Madsen JE (2007). Hemiarthroplasty or internal fixation for intracapsular displaced femoral neck fractures: randomised controlled trial. BMJ.

[7] Daisuke N, Takuji M, Hiromi H (2013). The treatment of femoral neck fractures: results using cephalocervical screws with sliding mechanism. Eur J Orthop Surg Traumatol.

[8] Ly TV, Swiontkowski MF (2008). Treatment of femoral neck fractures in young adults. Bone Joint Surg Am.

[9] Yih-Shiunn L, Chien-Rae H, Wen-Yun L (2007). Surgical treatment of undisplaced femoral neck fractures in the elderly. Int Orthop.

[10] Gregersen M, Krogshede A, Brink O, Damsgaard EM (2015). Prediction of reoperation of femoral neck fracture treated with cannulated screws in elderly patients. Geriatr Orthop Surg Rehabil.

[11] Singh M, Nagrath AR, Maini PS (1970). Changes in trabecular pattern of the upper end of the femur as an index of osteoporosis. J Bone Joint Surg Am.

[12] Lowe JA, Crist BD, Bhandari M, Ferguson TA (2010). Optimal treatment of femoral neck fractures according to patient’s physiologic age: an evidence-based review. Orthop Clin North Am.

[13] Schmidt AH, Asnis SE, Haidukewych G, Koval KJ, Thorngren KG (2005). Femoral neck fractures. Instr Course Lect.

[14] Spangler L, Cummings P, Tencer AF, Mueller BA, Mock C (2001). Biomechanical factors and failure of transcervical hip fracture repair. Injury.

[15] Stankewich CJ, Chapman J, Muthusamy R, Quaid G, Schemitsch E, Tencer AF (1996). Relationship of mechanical factors to the strength of proximal femur fractures fixed with cancellous screws. J Orthop Trauma.

[16] Yang JJ, Lin LC, Chao KH, Chuang SY, Wu CC, Yeh TT (2013). Risk factors for nonunion in patients with intracapsular femoral neck fractures treated with three cannulated screws placed in either a triangle or an inverted triangle configuration. J Bone Joint Surg Am.

[17] Gardner S, Weaver MJ, Jerabek S, Rodriguez E, Vrahas M, Harris M (2014). Predictors of early failure in young patients with displaced femoral neck fractures. J Orthop.

[18] Dolatowski FC, Adampour M, Frihagen F, Stavem K, Erik Utvåg S, Hoelsbrekken SE (2016). Preoperative posterior tilt of at least 20° increased the risk of fixation failure in Garden-I and -II femoral neck fractures. Acta Orthop.

